# Limited hatchery introgression into wild brook trout (*Salvelinus fontinalis*) populations despite reoccurring stocking

**DOI:** 10.1111/eva.12646

**Published:** 2018-06-14

**Authors:** Shannon L. White, William L. Miller, Stephanie A. Dowell, Meredith L. Bartron, Tyler Wagner

**Affiliations:** ^1^ Pennsylvania Cooperative Fish and Wildlife Research Unit Pennsylvania State University University Park Pennsylvania; ^2^ Department of Ecosystem Science and Management Pennsylvania State University University Park Pennsylvania; ^3^ U.S. Fish and Wildlife Service Northeast Fishery Center Lamar Pennsylvania; ^4^ U.S. Geological Survey Pennsylvania Cooperative Fish and Wildlife Research Unit Pennsylvania State University University Park Pennsylvania

**Keywords:** brook trout, captive stocking, introgression, *Salvelinus fontinalis*

## Abstract

Due to increased anthropogenic pressures on many fish populations, supplementing wild populations with captive‐raised individuals has become an increasingly common management practice. Stocking programs can be controversial due to uncertainty about the long‐term fitness effects of genetic introgression on wild populations. In particular, introgression between hatchery and wild individuals can cause declines in wild population fitness, resiliency, and adaptive potential and contribute to local population extirpation. However, low survival and fitness of captive‐raised individuals can minimize the long‐term genetic consequences of stocking in wild populations, and to date the prevalence of introgression in actively stocked ecosystems has not been rigorously evaluated. We quantified the extent of introgression in 30 populations of wild brook trout (*Salvelinus fontinalis*) in a Pennsylvania watershed and examined the correlation between introgression and 11 environmental covariates. Genetic assignment tests were used to determine the origin (wild vs. captive‐raised) for 1,742 wild‐caught and 300 hatchery brook trout. To avoid assignment biases, individuals were assigned to two simulated populations that represented the average allele frequencies in wild and hatchery groups. Fish with intermediate probabilities of wild ancestry were classified as introgressed, with threshold values determined through simulation. Even with reoccurring stocking at most sites, over 93% of wild‐caught individuals probabilistically assigned to wild origin, and only 5.6% of wild‐caught fish assigned to introgressed. Models examining environmental drivers of introgression explained <3% of the among‐population variability, and all estimated effects were highly uncertain. This was not surprising given overall low introgression observed in this study. Our results suggest that introgression of hatchery‐derived genotypes can occur at low rates, even in actively stocked ecosystems and across a range of habitats. However, a cautious approach to stocking may still be warranted, as the potential effects of stocking on wild population fitness and the mechanisms limiting introgression are not known.

## 1 INTRODUCTION

Supplementation of wild populations with captive‐raised individuals is an increasingly important management strategy for species of social, commercial, and recreational values (Araki, Cooper, & Blouin, 2007; Naish et al., 2007; Stowell, Kennedy, Beals, Metcalf, & Martin, 2015). With continued population declines in many fish species due to habitat loss, climate change, and historic overharvest, stocking programs have expanded to meet growing recreational demands and conservation goals (Araki & Schmid, 2010). Recreational stocking can provide an immediate increase in the size of the harvestable population and help to maintain populations that were formally sustained through natural reproduction. The maintenance of recreational opportunities for many species now largely relies on stocking enhancement programs (Askey, Parkinson, & Post, 2013), with some naturally reproducing populations comprised almost exclusively of individuals with captive ancestry (Evans & Willox, 1991; Ford et al., 2006).

Once released, captive fish, which tend to be larger and more aggressive than their wild counterparts (Huntingford, 2004), can prey upon juveniles from wild populations, compete for food, and usurp high‐quality habitats (Naish et al., 2007). However, captive fish often have low reproductive success, with relative fitness often less than half that of wild counterparts (Araki, Berejikian, Ford, & Blouin, 2008; Christie, Ford, & Blouin, 2014), and may be quickly removed from the environment through dispersal, harvest, or natural mortality (Baer, Blasel, & Diekmann, 2007). As such, recreational fish stocking can sometimes have small or no detectable effect on native fish communities (Weaver & Kwak, 2013).

Nonetheless, even with the potential for low survival and reproduction, recreational stocking still remains controversial due to the long‐term negative effects that captive individuals can have on wild populations (Utter, 2003; Weber & Fausch, 2003; Wollebæk, Heggenes, & Røed, 2010). In particular, successful interbreeding between hatchery and wild individuals, defined here as introgression, has the potential to threaten long‐term wild population viability by altering patterns of genetic diversity (Reisenbichler & McIntyre, 1977; Utter, 2003; Waples, 1991). Inbreeding, genetic drift, and unintentional and/or artificial selection in captive populations can increase the prevalence of genotypes with commercially valuable traits and alter allelic frequencies relative to wild populations. The phenotypes of captive individuals can confer a reduction in fitness relative to wild populations by altering the behavior, morphology, physiology, and timing of life history events (Ford et al., 2006; Naish, Seamons, Dauer, Hauser, & Quinn, 2013). Therefore, captive individuals, and their offspring, may not be successful in natural environments (Saikkonen, Kekäläinen, & Piironen, 2011; Stringwell et al., 2014; but see Allendorf et al., 2004 for a counterexample and discussion of heterosis).

Introgression can rapidly (i.e., in as little as one generation; Muhlfeld et al., 2009) modify wild population genetic diversity (Bowman et al., 2017; Ryman & Laikre, 1991), disrupt locally adapted gene complexes (Hallerman, 2003; Naish et al., 2007), homogenize genetic structure (Hindar, Ryman, & Utter, 1991; Marie, Bernatchez, & Garant, 2010), introduce maladaptive phenotypes into a population (Bolstad et al., 2017), disrupt expression of biologically relevant genes (Lamaze, Garant, & Bernatchez, 2013), and increase disease susceptibility (Currens et al., 1997). These genetic consequences of introgression, combined with reduced survival, reproduction, and competitive ability of introgressed offspring, can compromise population resiliency and future adaptive potential by reducing population sizes and eroding among‐population genetic variability (Tufto, 2017). Small, isolated populations may be particularly threatened by hatchery introgression due to limited gene flow (Ford, 2002; Lynch & O’Hely, 2001), which can increase the long‐term prevalence of domestic genotypes in wild populations.

Negative genetic consequences of stocking on wild populations have been documented in many fish species, particularly in commonly stocked species such as salmonids (Wollebæk et al., 2010). However, most studies to date that quantify hatchery introgression into wild populations have focused primarily on accidental releases from captive facilities and legacy effects of terminated stocking programs (see Araki & Schmid, 2010 for a review, and Kazyak, Rash, Lubinski, & King, 2018 for a large‐scale study on brook trout, *Salvelinus fontinalis*). Few studies have provided empirical estimates for introgression in populations managed with relatively long‐term (>10 years, in many cases) and recurring stocking events focused on population supplementation (but see Valiquette, Perrier, Thibault, and Bernatchez (2014) and Létourneau et al. (2018) for examples of lacustrine lake (*Salvelinus namaycush*) and brook trout populations). With repeated exposure to captive individuals, this common management scenario is predicted to induce high rates of introgression (Fleming & Petersson, 2001), particularly as the intensity and duration of stocking increase (Araguas, Sanz, & García‐ Marín, 2004). However, failure of stocked individuals to survive and reproduce, which may vary as a function of local and regional habitat features, could minimize rates of captive introgression. In this case, the genetic effects of stocking could vary considerably across the landscape. Therefore, a better empirical understanding of the relative rates of introgression in multiple stocked populations may help assess the potential long‐term effects of recreational stocking programs.

We quantified the degree of captive‐stock introgression in 30 wild brook trout populations and developed models to explore the correlation between introgression and local and regional habitat characteristics. Brook trout is a species of recreational value and high conservation concern throughout its native range in eastern North America. Despite ongoing efforts by state and federal agencies to restore populations, brook trout population size and geographic range have declined significantly in recent decades. Declines are largely due to instream habitat loss, deforestation, non‐native species introductions, and climate change (Hudy, Thieling, Gillespie, & Smith, 2008).

Because brook trout is a popular sport fishery in Pennsylvania, approximately 800,000 adult brook trout are stocked annually by the Pennsylvania Fish and Boat Commission (PFBC) and PFBC cooperative nurseries to increase recreational angling opportunities. Stocking is sometimes implemented on streams with existing wild brook trout populations and on streams adjacent to wild populations. As such, we predicted that introgression may occur in streams that are directly stocked and, because brook trout are capable of moving in excess of 10 km (Davis, Wagner, & Bartron, 2015), we also predicted introgression could occur in tributaries to stocked streams. Quantifying potential indirect effects of stocking is important because considerations for how hatchery stocking can influence wild populations are typically restricted to the stream of direct stocking efforts. However, movement of stocked individuals into nearby wild populations could result in unintended and unforeseen interactions between hatchery and wild trout that extend beyond the spatial scale of direct stocking efforts.

## 2 METHODS

### 2.1 Study area

**Figure 1:**
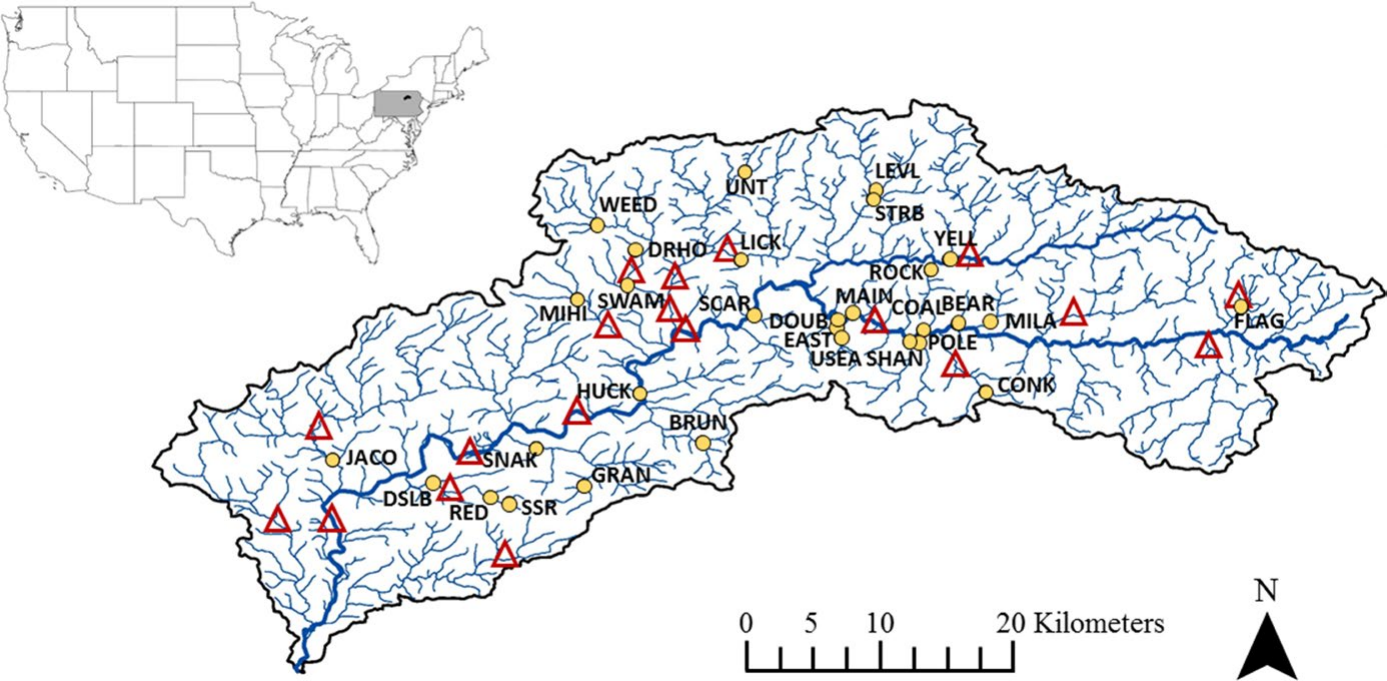
Distribution of 30 sample sites in the Loyalsock Creek watershed (circles) in northcentral Pennsylvania, the United States. The midpoint of stream sections stocked by the Pennsylvania Fish and Boat Commission (PFBC) and PFBC cooperative nurseries in 2015 is indicated by triangles. See Table 1 for full site names and sample sizes and Supporting Information Table S1 for detailed stocking histories

Loyalsock Creek is a 1,284 km^2^ watershed located primarily in Lycoming and Sullivan counties in northcentral Pennsylvania (Figure 1). This watershed is predominantly forested, and wild brook trout inhabit most tributaries to Loyalsock Creek. The mainstem of Loyalsock Creek is a large, warm/coolwater stream that is not thermally suitable for brook trout during summer, but is used seasonally for residence and migration.

The PFBC has maintained a recreational trout stocking program in Loyalsock Creek and select tributaries in the watershed for several decades. Because we sampled fish in 2015 (see *Sample Collection and Genotyping*), we examined stocking records from 2006 to 2015. Over this time period, 21 of our 30 study sites were either directly stocked or within 2 km of a stocking location. In streams that were stocked, stocking occurred once or twice per year from March to May. The average number of adult brook trout stocked per year at each site ranged from 151 to 3,491 at an average density of 125 fish/km (PFBC and cooperative nurseries combined, see Supporting Information Table S1 for more information). The average size of stocked fish was approximately 254–305 mm. In Pennsylvania, average wild adult brook trout density is 5 fish/100 m^2^ (Wagner, Deweber, Detar, Kristine, & Sweka, 2014) and average size of adult brook trout in Loyalsock Creek sampled during this study was approximately 120 mm. As such, brook trout density and biomass can increase by several orders of magnitude following a stocking event.

Only PFBC‐operated state fish hatcheries are used in PFBC stocking efforts, and the majority of fish stocked in the Loyalsock Creek watershed originate from the PFBC’s Tylersville State Fish Hatchery, with limited stocking from Benner Spring State Fish Hatchery. While captive fish are occasionally moved among hatcheries, wild fish have not been used to supplement hatchery broodstocks in Pennsylvania for at least 50 years (Brian Wisner, PFBC, personal communication). Sport fishing organizations also stock streams in the Loyalsock Creek watershed with fish from a PFBC cooperative nursery. The PFBC provides fish to its cooperative nurseries, and so genetic samples collected from PFBC hatcheries are representative of fish from cooperative nurseries. Private landowners sometimes stockfish from privately operated, non‐PFBC‐sponsored hatcheries. Records are not maintained for private stocking events, but they represent a minimal source of stocked fish in the watershed.

### 2.2 Sample collection and genotyping

Using backpack electrofishing, we collected between 2 and 197 (average = 58) tissue samples from adult brook trout (i.e., >100 mm; Whiteley et al., 2012) from each of 30 sites distributed throughout the Loyalsock Creek watershed from June 2015 to April 2017. In January 2017, tissue samples were obtained from 50 to 100 individuals from five brook trout strains at PFBC state fish hatcheries, including the Oswayo (OSW), Tylersville (TYL), and Bellefonte (BELL) strains and two representative groups from the Benner Springs strain (BNSPB, BNSPLP). For all fish, we excised a 5‐mm^2^ portion of the upper caudal fin and preserved the tissue in 95% nondenatured ethanol.

Genomic DNA was extracted for all samples following the manufacturers’ protocols for the MagBind^®^ Tissue DNA Kit KF (Omega Bio‐tek, Norcross, GA). All DNA extractions were carried out using the Kingfisher^®^ Flex (Thermo Scientific, Waltham, MA) automated purification system. Samples were genotyped at 12 microsatellite loci developed for use in brook trout, including S*fo*C‐113, S*fo*D‐75, S*fo*C‐88, S*fo*D‐100, S*fo*C‐129, S*fo*C‐24, S*fo*B‐52, S*fo*C‐28, S*fo*C‐38, S*fo*C‐79, S*fo*C‐86, and S*fo*D‐91 (King, Lubinski, Burnham‐Curtis, Stott, & Morgan, 2012).

Loci were combined into three multiplexes for polymerase chain reaction (PCR) amplification. Each 15 μl‐PCR consisted of 1.5 μl of genomic DNA extract along with 1.5× PCR buffer, 3.75 mM MgCl_2_, 0.3175 mM dNTPs, 0.08–0.18 μM of each forward and reverse primer, 0.08 units/μl of GoTaq Flexi DNA polymerase, and deionized water. The amplification cycle for all multiplex mixes consisted of an initial denaturation at 94°C for 2 min, followed by 35 cycles of: 94°C denaturation for 45 s, 56°C annealing for 45 s, and 72°C extension for 2 min. A final extension was conducted at 72°C for 10 min.

An Applied Biosystems 3130xl genetic analyzer (Thermo Fisher Scientific) was used for fragment analysis. Alleles were individually scored using GeneMapper^®^ version 4.1 software (Thermo Fisher Scientific) by two independent readers. Genotypes were obtained for >99% of loci across all individuals. For purposes of quality assurance, we re‐extracted and genotyped 10% of all samples and compared the results to those of original samples. The genotyping error rate was <1% per locus.

Because some sites were sampled more than once, we used the program Cervus 3.0.7 (Kalinowski, Taper, & Marshall, 2007) to identify duplicate genotypes. The probability of two unique individuals sharing the same multilocus genotype was <0.0001%, and so duplicate genotypes were considered repeated samples and removed from the analysis. In total, we sampled 1,742 unique wild and 300 hatchery brook trout (Table 1). For sites with repeat sampling, we combined all samples into the same population. As we quantified assignment probabilities independently for each fish without respect to population origin, combining samples does not affect the outcome of our analysis.

**Table 1:** Site name abbreviations, sample sizes, measures of population genetic diversity (N_A_—average number of alleles per locus, H_E_‐Nei’s unbiased estimate of heterozygosity), and average stocking densities for 30 wild populations and five hatchery strains of brook trout used to determine the degree of introgression in the Loyalsock Creek watershed

Site name	Abbr.	Sample Size	N_A_	H_E_	Average *p*(wild)	Number of Wild Fish	Number of Introgressed Fish	Number of Hatchery Fish	Average Stocking Density (fish/km)
Hatchery									
Bellefonte	BELL	100	7.00	0.69	0.11	0	38	62	
Oswayo Hatchery, Oswayo Strain	OSW	50	6.17	0.69	0.04	0	10	40	
Benner Springs, Lighthouse Strain	BNSPLP	50	3.33	0.56	0.00	0	0	50	
Oswayo Hatchery, Tylersville Strain	TYL	50	3.33	0.54	0.00	0	0	50	
Benner Springs, B Strain	BNSPB	50	3.33	0.55	0.00	0	0	50	
Wild									
Mill Run‐ Laporte	MILA	50	3.92	0.54	1.00	50	0	0	
Level Run	LEVL	50	8.17	0.72	1.00	50	0	0	
Mainstem Loyalsock Creek^a^	MAIN	2	2.83	0.79	1.00	2	0	0	39.76
Upstream East Branch	EAST	50	4.67	0.57	0.99	50	0	0	
Unnamed Tributary to Elk Creek	UNT	48	7.08	0.70	0.99	48	0	0	
Bear Run^b^	BEAR	50	6.33	0.69	0.99	50	0	0	39.76
Streby Run	STRB	50	7.92	0.74	0.99	49	1	0	
Huckle Run^b^	HUCK	22	3.92	0.58	0.99	22	0	0	31.48
Yellow Run^b^	YELL	50	5.67	0.68	0.99	50	0	0	126.56
Sand Spring Run^b^	SSR	50	7.33	0.73	0.99	50	0	0	528.97
Grandad Run	GRAN	50	7.50	0.76	0.99	49	1	0	
Red Run^b^	RED	50	7.25	0.72	0.99	49	1	0	528.97
Pole Bridge Run^a^^,^ b	POLE	109	7.42	0.69	0.99	106	3	0	77.67
Scar Run^b^	SCAR	50	5.67	0.68	0.99	48	2	0	31.48
Downstream East Branch^b^	DSEA	73	7.92	0.69	0.99	70	3	0	107.56
Shanerburg Run^a^^,^ b	SHAN	197	9.92	0.73	0.99	192	5	0	81.16
Coal Run^b^	COAL	50	6.25	0.68	0.98	48	2	0	39.76
Brunnerdale Run	BRUN	50	7.00	0.75	0.98	47	3	0	
Rock Run^b^	ROCK	50	5.58	0.68	0.98	46	4	0	126.56
Snake Run^b^	SNAK	50	5.17	0.60	0.98	45	5	0	76.34
Weed Run^b^	WEED	50	7.08	0.64	0.98	47	3	0	251.69
Dry Run‐ Hoagland Branch^b^	DRHO	50	6.33	0.66	0.98	47	3	0	251.69
Jacoby Hallow^b^	JACO	50	7.42	0.72	0.97	49	0	1	36.42
Lick Run^b^	LICK	50	5.83	0.72	0.96	42	8	0	32.03
Double Run^a^^,^ b	DOUB	154	8.83	0.74	0.95	129	25	0	74.75
Flag Marsh Run	FLAG	49	7.67	0.72	0.95	40	9	0	
Mill Creek‐Hillsgrove^a^	MIHI	50	7.67	0.71	0.95	46	2	2	65.59
Conklin Run	CONK	50	4.33	0.63	0.94	36	14	0	
Swamp Run^b^	SWAM	50	7.83	0.72	0.89	44	1	5	251.69
Little Bear Creek^a^^,^ b	DSLB	38	8.00	0.76	0.77	28	2	8	353.49

*Note*. Records are sorted by descending values of *p*(wild). Average *p*(wild) and number of individuals that assigned to each class were determined by individual assignment to one of two (hatchery and wild) simulated populations. See supplementary text for more detailed stocking records.

a Stocking occurs at the sample location.

b Stocking within 2 km of the sample location.

### 2.3 Simulating population centroids

Differences in sample size and allelic richness, which were both highly variable across the populations sampled (Table 1), have been shown to bias individual estimates of assignment probability using traditional algorithms found in the programs STRUCTURE and GeneClass (Halbisen & Wilson, 2009; Wang, 2017). In particular, there is a tendency for algorithms to bias assignment toward populations of larger sample size and higher genetic diversity. To minimize these effects, we quantified each individual’s probability of wild origin, *p*(wild), following the methods of Karlsson, Diserud, Moen, and Hindar (2014). The method described by Karlsson et al. (2014) circumvents the aforementioned challenges by simulating centroids for the wild and hatchery populations that represent average allele frequencies across all populations, and uses these centroids, rather than putative sample populations, as the basis for individual fish assignment.

Provided that there is significant differentiation in wild and captive populations (an assumption we tested, as described below), restricting assignment to either hatchery or wild origin is a more powerful analysis that results in less ambiguous assignment probabilities. This is particularly advantageous when there is at least intermittent gene flow among populations or between groups (i.e., hatchery and wild), as traditional approaches may fail to reach assignment consensus when probabilities are divided among several populations. Further, because centroids represent the average allele frequencies of populations within the same group, this dichotomous classification scheme minimizes spurious assignments related to inexhaustive sampling of potential source populations. This was particularly important for this analysis, as it was not possible to sample hatcheries used for private stocking or all streams in the Loyalsock Creek watershed.

Wild and hatchery population centroids were constructed from 22 randomly selected fish from each wild population and 50 randomly selected fish from each hatchery population. Sample size was based on the smallest reasonable population sample size within each group. Only two individuals were captured from mainstem Loyalsock Creek, and so fish from that site were not used to generate population centroids. Sampling was performed without replacement, and the randomly sampled wild and hatchery populations consisted of 638 and 250 unique fish, respectively. Using the program HybridLab (Nielsen, Bach, & Kotlicki, 2006), we simulated mating events within the randomly selected wild and hatchery populations to obtain 500 simulated wild and 500 simulated hatchery individuals. These simulated individuals comprised the wild and hatchery centroids.

To evaluate the precision of the centroid generation method, we constructed ten unique wild and hatchery centroids, each time using a different set of randomly selected individuals. Average within‐group *F*_ST_ for both wild and hatchery centroids was <0.001 (variance <0.00001) and average *F*_ST_ between hatchery and wild centroids was 0.07. This demonstrates that assignment probabilities and classification are unlikely to be affected by the centroid generation process. In addition, HybridLab conditions random mating on observed allele frequencies, thus ensuring that centroids are centered within the observed wild and hatchery populations and representative of average within‐group allelic variance. However, we checked this assumption with a principal coordinate analysis (PCoA) of pairwise *F*_ST_ and tested for conformance to Hardy–Weinberg expectations in GenAlEx 6.5 (Peakall & Smouse, 2006; Peakall & Smouse, 2012).

Using nonwild genotypes to generate the wild centroid could mischaracterize the wild centroid and bias individual assignments. At last, because the majority of individuals in our study assigned to wild origin (see *Results: Population Assignments of Sampled Fish*), the relative abundance of wild trout relative to hatchery or introgressed supplants the influence of nonwild genotypes in the centroid. However, we evaluated this assumption by generating ten centroids using only individuals from populations where all fish assigned to wild origin. Average *F*_ST_ between these centroids and the ten centroids generated from the full dataset was 0.003, which is an order of magnitude lower than the average *F*_ST_ among wild populations of brook trout in this study (0.07) and most wild brook trout populations reported in the literature (*e.g.,* Kelson, Kapuscinski, Timmins, & Arden, 2015). Because using more individuals to generate the wild centroid helps minimize assignment bias by increasing the amount of explained genetic variation in our samples, we elected to use the centroid generated from the full dataset for individual assignments. However, individual assignments with the more restrictive centroids would be nearly identical to the centroids generated from the full dataset.

### 2.4 Estimating individual wild probability

We estimated *p*(wild) for each sampled wild and hatchery individual using the program STRUCTURE v 2.3.4 (Pritchard, Stephens, & Donnelly, 2000) executed through the ParallelStructure package in R (Besnier & Glover, 2013). To minimize assignment biases due to unequal sample sizes (Kalinowski 2011), a separate STRUCTURE analysis was completed for each observed individual. Each analysis included the 500 simulated wild fish and 500 simulated hatchery fish (i.e., the population centroids), as well as the genotype for one sampled individual. For each STRUCTURE run, we applied 50,000 repetitions as burn‐in and 100,000 repetitions after burn‐in with no a priori information on sample location and assumed two populations (*K* = 2), which corresponded to the dichotomy between wild and hatchery populations. The coefficient of ancestry (*q*) to the wild cluster was interpreted as the *p*(wild) for each observed individual.

### 2.5 Assignment of individuals to wild, hatchery, or introgressed origin

To identify introgressed individuals, we needed to determine upper and lower thresholds for *p*(wild) commensurate with interbreeding between a wild and hatchery fish. To accomplish this, we used HybridLab to simulate 500 wild‐hatchery matings using the 638 wild and 250 hatchery fish randomly selected above. We then completed 500 independent runs of STRUCTURE, this time including the 500 simulated wild fish, 500 simulated hatchery fish, and one simulated wild‐hatchery cross.

We developed an expected distribution for *p*(wild) for introgressed individuals using the 500 *q*‐values from the STRUCTURE analysis of the simulated wild‐hatchery crosses. Values of *p*(wild) that fell between the 2.5 and 97.5 percentile were considered characteristic of introgressed origin, and observed wild and hatchery individuals with a *p*(wild) that fell within this range were classified as introgressed. Fish with a *p*(wild) below the 2.5 percentile were classified as pure hatchery origin, and fish with a *p*(wild) above the 97.5 percentile were classified as pure wild origin. Others have recommended different methods for identifying introgressed individuals, including the use of 5th and 95th percentiles (Karlsson et al., 2014) and raw STRUCTURE *q*‐values between 0.10 and 0.90 (Harbicht, Alshamlih, Wilson, & Fraser, 2014; Harbicht, Wilson, & Fraser, 2014; Vähä & Primmer, 2006). However, we elected to use a larger interval to characterize introgressed *p*(wild) because it offers a more powerful estimate of introgression and is more likely to assign individuals as introgressed rather than originating from a hatchery or wild source.

Because the true ancestry of wild‐caught individuals is unknown, the accuracy of our assignment method could not be evaluated using the empirical data. Therefore, we repeated the analysis on a simulated dataset with a known number of introgressed individuals. This analysis also enabled us to evaluate the accuracy of individual assignments when using different assignment thresholds for classifying introgression (i.e., using a narrower range than the 2.5 and 97.5 percentiles). Our method detected introgressed individuals with zero error when using the 2.5 and 97.5 percentiles. However, error rate increased to 30% when using the 5 and 95 percentiles as assignment thresholds (see Supporting Information Methods S1 for more details).

Using a larger range of values of *p*(wild) to characterize introgression increases the likelihood of assigning individuals to admixed origin, which also likely increases the ability to detect post‐F1 introgression. To evaluate whether our methods were capable of detecting post‐F1 introgression, we conducted a parallel analysis using the program NewHybrids (Anderson & Thompson, 2002) to determine the probability that a sampled fish was from wild, hatchery, first‐generation, or post‐first‐generation origin (representing the cumulative probability of F2 or backcross). Assignment probabilities were determined by running 100,000 sweeps of four chains after 25,000 burn‐in sweeps with a Jeffrey’s prior for θ and π.

### 2.6 Environmental correlates to introgression

We used a hierarchical logistic regression to model the probability of a wild‐caught fish assigning as introgressed as a function of several environmental covariates. Wild‐caught fish that assigned to a putative hatchery origin were removed prior to analysis. Environmental covariates included site‐level predictors that described local habitat and water quality (temperature, dissolved oxygen, conductivity, hardness, total alkalinity, pH, stream width, and adult brook trout density calculated with the Zippkin (1958) removal method [J.M. Niles, unpublished data, Susquehanna University Freshwater Research Initiative]). We also included watershed‐level predictors that were derived from Geographic Information Systems (watershed area [km^2]^, distance to closest stocking reach, and land use). To account for environmental stochasticity, which could alter the interannual probability of introgression, we averaged site‐level data from 2013 to 2015 (see Supporting Information Table S2 for the average and range of values for each covariate). To avoid issues associated with multicollinearity, highly correlated (absolute value of Pearson’s *r* > 0.7) variables were not included in the same model. We also excluded land use because the majority of sites occurred in a state forest, and difference in land use among watersheds was not biologically meaningful (*e.g.,* all but one site had a watershed with >90% forest cover). All covariates were transformed into *z*‐scores prior to analysis.

We fitted four candidate models to evaluate hypotheses about potential environmental correlates to introgression. The multiscale model included all site‐ and watershed‐level predictors and represented the hypothesis that introgression is mediated by habitat at multiple scales. The second model included only site‐level covariates and represented the hypothesis that site‐level factors are the dominant environmental predictors of introgression. In contrast, the third model included only watershed‐level covariates and was used to evaluate the hypothesis that large‐scale habitat features are more important than local properties in predicting introgression. The fourth model, the literature‐supported model, was similar to the multiscaled model, but only included covariates that have been previously identified in the literature as being important predictors of introgression in salmonids (Harbicht, Alshamlih, et al., 2014; Harbicht, Wilson, et al., 2014; Marie, Bernatchez, & Garant, 2012; Splendiani, Ruggeri, Giovannotti, & Caputo, 2013). All models included sample site as a random effect to account for the fact that there were multiple individuals collected from the same site, and we would expect genotypes from individuals collected within a site to be more similar than genotypes from individuals among sites (see Supporting Information Table S3 for model descriptions).

Models were executed using the lme4 package in R (Bates, Maechler, Bolker, & Walker, 2014), and models were compared using an information theoretic approach to compare competing hypotheses (Burnham & Anderson, 2002). In particular, we calculated the ΔAIC_C_ and Akaike weights (*w*_*i*_) for each model.

## 3 RESULTS

### 3.1 Population centroids

Wild populations were genetically more diverse than hatchery populations with an average of 6.62 alleles per locus (*SE* = 0.16) and expected heterozygosity of 0.68 (*SE* = 0.01) compared to 4.63 alleles per locus (*SE* = 0.28) and an expected heterozygosity of 0.60 (*SE* = 0.02) in hatchery populations (Table 1). There was significant genetic differentiation between observed wild and hatchery populations. Average *F*_ST_ within hatchery and wild populations was 0.06 (range: 0.01–0.08) and 0.07 (range: 0.01–0.16), respectively, and average *F*_ST_ among hatchery and wild populations was 0.13 (range 0.06–0.22). All pairwise *F*_ST_ values were significant at *p < *0.001.

**Figure 2:**
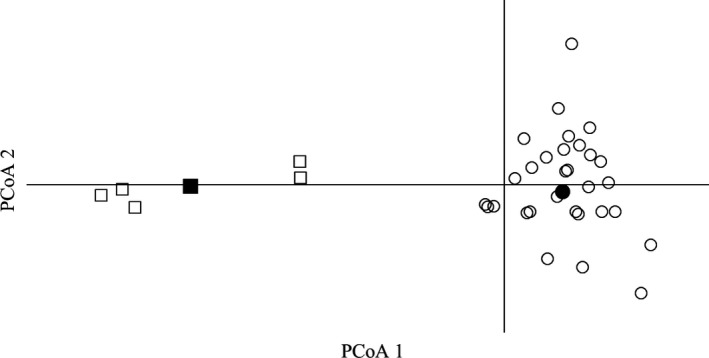
PCoA plot for pairwise *F*_ST_ estimates between sampled wild (open circles) and hatchery brook trout populations (open squares). Simulated wild and hatchery population centroids are shown in closed circles and squares, respectively. The first PCoA axis explained 28.6% of total sample variance

**Figure 3:**
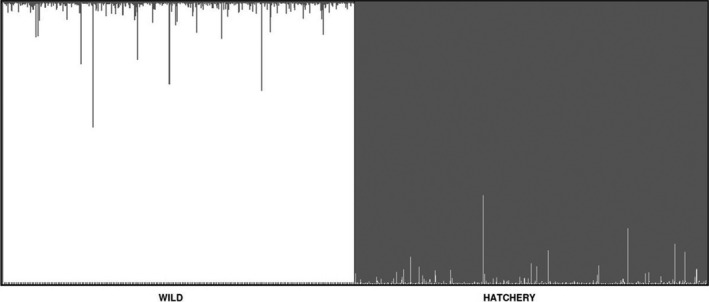
Bar plot representing the classification of simulated hatchery and wild individuals to putative centroid populations in STRUCTURE. Colors within bars represent the probability of each simulated individual assigning to either a wild cluster (white) or captive cluster (gray). No simulated individuals were incorrectly assigned to the competing cluster and few exhibited significantly admixed genotypes

Samples from wild and hatchery populations were separated most significantly by Axis 1 on the PCoA, which explained 28.6% of the total variance in the dataset. Axis 2 did not result in separation between the groups and only explained 9.2% of the total variance. Simulated wild and hatchery population centroids were located in the middle of their respective populations on the PCoA (Figure 2) and were distinguishable in STRUCTURE (Figure 3) with no individuals incorrectly assigning to the competing cluster. Both centroid populations met the expectations of Hardy–Weinberg equilibrium (*p > *0.015 at all loci, with Bonferroni‐corrected α = 0.002).

### 3.2 Population assignments for sampled fish

**Figure 4:**
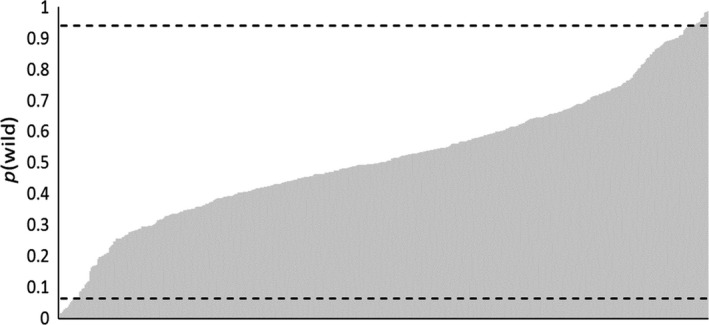
Distribution for probability of wild descent for 500 simulated introgressed individual brook trout. Estimates were generated by analyzing the multilocus genotype of each individual fish in STRUCTURE along with simulated wild and hatchery centroid populations. Horizontal dashed lines represent the lower 2.5 and upper 97.5 percentiles. Sampled individuals that had a wild probability below the 2.5 percentile were assigned to hatchery origin, and individuals above the 97.5 percentile were assigned to wild origin. Sampled individuals with *p*(wild) between these cutoff values were assigned to introgressed origin

The expected distribution of *p*(wild) for an introgressed fish had a lower 2.5 percentile of 0.06 and an upper 97.5 percentile of 0.94 (Figure 4). Therefore, when determining individual assignments, a fish that had a *p*(wild) between 0.06 and 0.94 was classified as introgressed. Fish with a *p*(wild) that fell below or above this range were classified as hatchery and wild origin, respectively.

**Figure 5:**
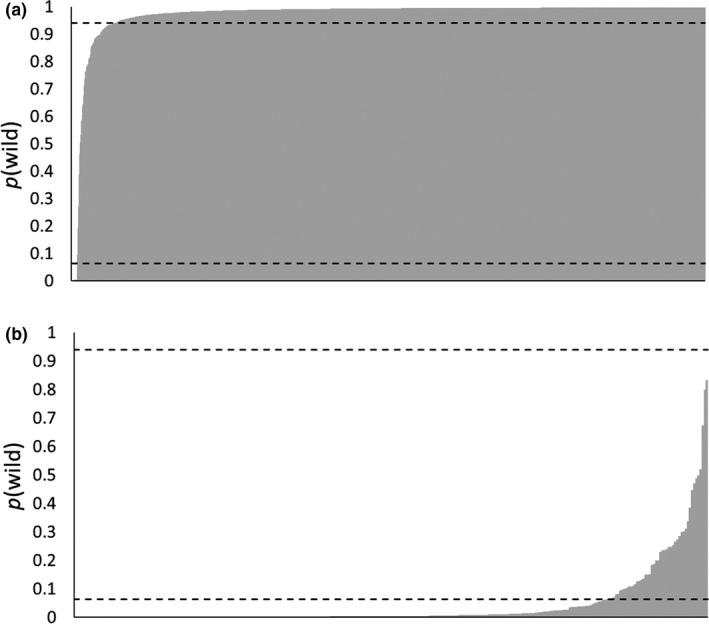
Distribution of wild probabilities *p*(wild) for 1,742 wild‐caught (a) and 300 hatchery (b) brook trout, sorted by ascending *p*(wild). Estimates were generated by analyzing the multilocus genotype of each individual fish in STRUCTURE along with simulated wild and hatchery population centroids. Horizontal dashed lines represent the lower 2.5 and upper 97.5 percentiles from the null distribution for wild probability for an introgressed individual (see Figure 3)

**Figure 6:**
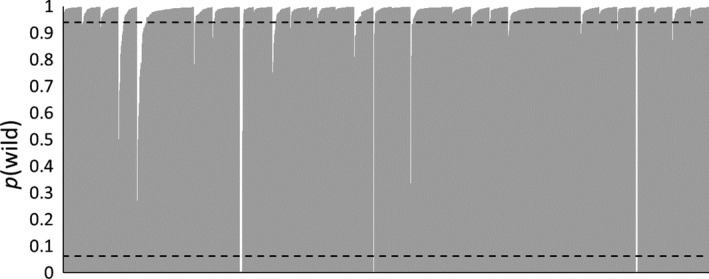
Wild probabilities (*p*(wild), shown in grey) for 1,742 wild‐caught brook trout. Fish are sorted by population and in ascending order of *p*(wild) within a population. Populations appear in the same order as Table 1. Horizontal dashed lines represent the lower 2.5 and upper 97.5 percentiles from the null distribution for wild probability for an introgressed individual (see Figure 3)

Of the 1,742 wild‐caught fish sampled, 16 (<1%) assigned to pure hatchery origin and 97 (5.6%) to introgressed origin (Figure 5a). Average *p*(wild) for all wild‐caught fish was 0.97; however, there was considerable variation in average *p*(wild) across populations (Figure 6). There were 23 populations with average *p*(wild) >0.97, and only two populations had an average *p*(wild) <0.90, including SWAM (0.89) and DSLB (0.77). For both sites, low average *p*(wild) was due to the presence of multiple fish assigning to hatchery origin. Of note, DSLB is the site with highest stocking densities and, while only two fish assigned as introgressed, we did detect eight fish of pure hatchery origin. Sites that had the largest proportion of introgressed individuals included CONK (28%) and FLAG (18%). However, there were 20 populations comprised of <5% introgressed individuals, with 10 populations having no fish assigning as introgressed.

Of the 300 hatchery fish sampled, 252 (84%) classified as pure hatchery origin and 48 (16%) as admixed origin (Figure 5b). No individual sampled from a hatchery assigned to pure wild origin. All hatchery strains stocked in the Loyalsock Creek watershed (BNSPLP, BNSPB, and TYL) had an average *p*(wild) < 0.005, and all fish from those strains assigned as pure hatchery origin.

All hatchery fish that assigned as introgressed were from the OSW (average *p*(wild) 0.04) and BELL (average *p*(wild) 0.11) strains. Wild fish have not been used in Pennsylvania hatcheries for at least 50 years, and so we know that these fish are not the product of wild‐hatchery introgression. A more likely explanation is that hatchery fish that assigned as introgressed are the offspring from two different hatchery strains, at least one of which more genetically similar to wild populations than the hatchery strains used in our analysis. Unlike TYL and BNSP, hatcheries containing the OSW and BELL strains receive excess hatchery fish from across Pennsylvania at the end of each stocking season and subsequently use those fish as broodstock (Brian Wisner, PFBC, personal communication). Our study only characterized fish from four of 13 state fish hatcheries (and each state fish hatchery can maintain multiple unique strains). Thus, although ambiguous genotypes could have resulted in the incorrect assignments, a more likely scenario is that introgressed hatchery fish are the result of unexplained genetic variation from unsampled hatchery strains. At last, fish from the OSW and BELL strains are not stocked at, or near, our sample sites by PFBC, so these assignment errors do not influence the accuracy of our results. However, the OSW and BELL strains were retained in our analysis to help account for uncertain sources of stocked fish used in undocumented, private stocking events.

There was consensus between NewHybrids and STRUCTURE for 1933 of 2,042 individuals (95%). NewHybrids assigned 52 individuals as introgressed, all to post‐F1 origin. STRUCTURE assigned 41 of these individuals (79%) as introgressed and 11 to hatchery origin. The 11 fish that assigned as introgressed by NewHybrids but to hatchery origin by STRUCTURE were from one of the hatchery strains (thus representing an assignment error by NewHybrids).

Of the remaining 98 discrepancies, 36 individuals (37%) had inconclusive ancestry in NewHybrids (i.e., <80% assignment probability to any one category, Sloss, Jennings, Franckowiak, & Pratt, 2008), and 61 individuals (62%) were assigned as wild by NewHybrids but introgressed in STRUCTURE. Only one individual was assigned to hatchery in NewHybrids and introgressed in STRUCTURE. This suggests that, while the two methods provided similar results, the STRUCTURE simulation method offers a more conservative and, in the case of hatchery individuals, more accurate, estimate of introgression. We elected to make inferences about individual population origin based on the STRUCTURE simulation method, as STRUCTURE is a more powerful algorithm when using fewer loci (Vähä & Primmer, 2006) and because identification of cohort structure was not necessary to accomplish the objectives of this analysis. However, it is worth noting that NewHybrids assigned all individuals to post‐F1 origin, suggesting that our STRUCTURE method was likely capable of detecting post‐F1 introgression.

### 3.3 Environmental correlates to introgression

The two top‐ranked models, the watershed‐level model and the site‐level model, were nearly indistinguishable based on ΔAIC_C_ (ΔAIC_C_ = 0.35) with weights of 0.48 and 0.40, respectively. There was very little support for either the multiscaled (*w*_*i*_ = 0.07) or literature‐supported (*w*_*i*_ = 0.05) models. All models explained very little among‐site variation in introgression, with *R* ^2^ < 0.03 for all models (Supporting Information Table S3).

Given that the watershed‐level and site‐level models had similar support, we averaged these two models to generate a consensus model. The 95% confidence intervals for all parameters in the consensus model overlapped zero (see Supporting Information Table S4 for parameter estimates and standard errors).

## 4 DISCUSSION

Supplemental stocking with captively propagated individuals is frequently practiced to increase recreational opportunities (Naish et al., 2007) and mitigate declines of threatened and endangered populations (Fraser, 2008). Frequent stocking is predicted to increase the incidence of captive introgression into wild populations (Fleming & Petersson, 2001); however, few studies have evaluated the prevalence of introgression in actively stocked populations. After sampling 30 wild brook trout populations, many of which at or near recent stocking locations, we found low incidences of introgression. Less than 6% of all wild‐caught fish probabilistically assigned to admixed origin, and, on average, 94% of all fish within each sampled population assigned to pure wild origin. Our results suggest that the risk of domestic introgression in ecosystems with a history of repeated stocking may be minimal for some species and populations.

Introgression can alter native population genetic diversity, disrupt the frequency of locally adapted gene complexes, and decrease population fitness (Naish et al., 2007). However, our understanding of the probability of these events occurring is based largely on studies of unintentional releases, legacy effects of terminated stocking practices, and effects of large‐scale fishery augmentation projects (Fleming & Petersson, 2001; Ford et al., 2006; Naish et al., 2007). While these studies provide valuable insights, they may not be adequate surrogates for direct study of present‐day sport fish stocking programs. Many documented instances of accidental releases occur in relatively large habitat patches, such as estuaries and bays, where rapid dispersal of escaped individuals away from the source can increase survival and reproduction (Piccolo & Orlikowska, 2012). On the contrary, stream salmonids are not as vagile and are primarily constrained to low‐order watersheds (Kanno, Vokoun, & Letcher, 2011) where increased angling pressure may lead to higher harvest rates, particularly for domestic fish which are often more susceptible to angling (García‐Marín, Sanz, & Pla, 1999). Headwater streams are also spatially and temporally complex systems that are prone to rapid changes in flow, temperature, and food availability. This stochasticity may exacerbate the phenotypic mismatch of hatchery fish with wild environments, thereby further decreasing fitness and survival of hatchery individuals in the wild.

Other studies of domestic introgression focus on populations that were last stocked over 20 years ago, when broodstocks had been held in captivity for fewer generations. Although significant genetic change can occur over short time spans (Christie et al., 2014), minimal genetic differences between historic hatchery and wild stocks are not expected to significantly influence patterns of genetic diversity in present‐day wild populations (Ford, 2002; Naish et al., 2007; but see Stowell et al., 2015 for a counterexample in cutthroat trout (*Oncorhynchus clarkii*) populations).

A significant limitation with studies of historic and accidental releases is that power to detect introgression decreases with generation time (Vähä & Primmer, 2006). Assuming wild and hatchery individuals are genetically distinct (an assumption that is not always met when studying historic populations, as discussed above), first‐generation hybrids can be readily detected using relatively simple genetic assignment tests. However, post‐F1 offspring can be difficult to identify without multiple diagnostic loci or comparisons to reference samples collected before captive release (Madiera, Gómez‐Moliner, & Barbé, 2005; Vähä & Primmer, 2006). In the same way, backcrosses with wild or captive individuals and variability in allelic inheritance can confound the expected distribution of ancestry coefficients, which makes assignment to any specific cohort difficult without a large number (>24) of informative loci (Vähä & Primmer, 2006). These factors can inflate variance estimates and limit the ability to detect introgression, even at the first‐generation level (Karlsson et al., 2014).

We circumvent these challenges by studying populations that are currently stocked, which should have a large proportion of first‐generation introgressed offspring relative to other cohort classes if interbreeding between stocked and wild fish is occurring. While we base assignment thresholds from only first‐generation simulated crosses, the use of conservative thresholds for individual assignments provided high power to detect introgression beyond the F1 cohort (as determined via independent analysis in NewHybrids). Thus, our assignment methods present an improvement over other methods (Vähä & Primmer, 2006) and suggest that single crosses, rather than more extensive simulation of additional cohorts, are sufficient to identify contemporary introgression. This method is particularly advantageous for detecting introgression given that simulations beyond the first generation become intractable, and the power to assign individuals to specific cohorts declines as the distribution of assignment values begins to overlap among groups. Attempts to simulate beyond the first generation are further complicated for species like brook trout, where overlapping generations will increase the variance of expected distributions and decrease assignment power.

The power of the analysis draws from the generation of an expected distribution for introgressed *p*(wild), which is favored in a logistical sense because it does not require historic reference samples. Using this distribution as the basis for population assignment removes uncertainty associated with interpretation of *q*‐values from STRUCTURE output (Van Wyk et al., 2016). Further, it allows researcher to adjust assignment thresholds to balance the trade‐off between conservatism and bias when estimating introgression in their study system. For example, a larger range of *q*‐values used to classify introgression may be more conservative, but could introduce bias by misclassifying wild or hatchery individuals as introgressed. The significance of this trade‐off will likely depend on the research and conservation objectives.

The analysis further gains power by restricting assignment to two population centroids, rather than putative sample populations, which decreases assignment errors by avoiding assumptions of assignment algorithms and minimizing bias associated with sampling design (Karlsson et al., 2014; Wang, 2017). In particular, this approach negates the need to exhaustively sample wild and hatchery populations, provided that samples are representative of total within‐population variation (Karlsson et al., 2014). Application of this method to simulated data here (Supporting Information Methods S1) and by Karlsson et al. (2014) using populations of comparable genetic differentiation as our observed wild and hatchery populations suggests that this method is a reliable alternative for individual assignment tests.

As one of the most commercially and recreationally valuable fish taxa, captive releases of salmonid species have been occurring for nearly 150 years. As such, salmonids have been the focal species in the majority of studies of introgression in fish and wildlife populations (Araki & Schmid, 2010). Many of these studies have produced equivocal results, even when limiting comparisons within a single species. For example, farmed Atlantic salmon (*Salmo salar*) unintentionally released into large rivers have shown a high propensity to interbreed with native populations and disrupt wild population genetics, while other populations invaded by captive escapees showed limited introgression (Glover et al., 2013; Hansen, Fraser, Meier, & Mensberg, 2009; Ozerov et al., 2016). Likewise, studies of fluvial brown trout (*Salmo trutta*) and lacustrine lake trout and brook trout suggest that rates of introgression vary across a landscape, even when populations are separated at small spatial scales (Almodóvar, Nicola, Elvira, & García‐Marín, 2006; Halbisen & Wilson, 2009; Madiera et al., 2005; Marie et al., 2012).

Our results corroborate those by Humston et al. (2012) and suggest variable, but overall lower degrees of introgression than other studies of stream salmonids (see Fleming & Petersson, 2001 for comparison). Of note, all sites comprised of >10% introgressed individuals are not directly stocked by PFBC or cooperative nurseries, including one site (CONK) which has no record of stocking for over 50 years and is isolated by a downstream impoundment. While the possibility of private stocking cannot be dismissed, this result suggests that there may be legacy effects of genetic introgression that continue to accumulate in populations long after the termination of management activities. In addition, while natural selection may be able to purge deleterious functional genes within several generations (Harbicht, Alshamlih, et al., 2014; Harbicht, Wilson, et al., 2014), introgression may remain detectable in noncoding regions of the genome (Tufto, 2017).

Why the degree of introgression varies significantly across species and among populations remains uncertain. Some have suggested a negative correlation between the prevalence of introgression and population size (Harbicht, Alshamlih, et al., 2014; Harbicht, Wilson, et al., 2014). This is presumably because competition for limited resources is higher in populations that approach carrying capacity, which decreases the probability of domestic fish surviving and reproducing in the wild. This hypothesis has support from studies that have found higher rates of introgression in populations inhabiting high‐elevation and/or low pH environments, both of which are known to limit brook trout productivity (Harbicht, Alshamlih, et al., 2014; Harbicht, Wilson, et al., 2014; Marie et al., 2012; Splendiani et al., 2013).

Results of our models correlating introgression to environmental predictors explained little variation in introgression among populations. This was not unexpected given that introgressed individuals constituted <6% of our sample, and so there was minimal among‐site variability to model. However, even though the estimated effects for both site‐ and watershed‐level predictors had large uncertainty, the direction of the estimated effects generally agreed with published literature. For example, effect estimates were negative for pH and adult density and positive for temperature. This corroborates findings from others, who have found empirical evidence that introgression was more common in lakes with low pH, high temperature, and smaller adult densities (Harbicht, Alshamlih, et al., 2014; Harbicht, Wilson, et al., 2014; Létourneau et al., 2018; Marie et al., 2012). Likewise, the predicted direction for parameter estimates for stream width and watershed area were both negative, indicating the potential for decreased introgression in larger stream reaches. As smaller streams are characterized by stochasticity at finer temporal and spatial scales, this result could support findings from Splendiani et al. (2013) that suggest increased introgression at sites with more unstable stream flows. Given the overall uncertainty of our parameter estimates, future work would benefit from more direct study of introgression in stream ecosystems to determine the effect of habitat on stocked fish survival and reproduction.

The variable fitness of stocked individuals may also explain why introgression differs across studies. High mortality, low‐quality gametes, and low offspring survival are typical of captive individuals released into wild populations (Araki & Schmid, 2010), particularly as generation time in captivity increases. This can be due to reductions in genetic diversity and selection for genotypes and phenotypes adapted to the hatchery environment (Naish et al., 2007), which may not be optimal for persistence in the wild. While we did not explicitly examine individual survival and reproduction, few hatchery brook trout are collected during summer surveys by PFBC staff (Jason Detar, PFBC, personal communication), suggesting limited long‐term survival of brook trout stocked in the Loyalsock Creek watershed.

While there was minimal introgression at any single site, 21 sites had at least one wild‐caught individual assign to either introgressed or hatchery origin. In some cases, hatchery influence was detected in populations located several kilometers from the nearest stocking location and with no history of previous stocking. Domestic trout can readily disperse after release (Bettinger & Bettoli, 2002), particularly in spring when trout can use mid‐reach rivers (which often exceed trout thermal tolerance in summer, Aunins, Petty, King, Schilz, & Mazik, 2015) as movement corridors. While mortality is often higher for more mobile, stocked fish (Aarestrup, Jepsen, Koed, & Pedersen, 2005), our results suggest that stocked trout may move into, and survive in, wild populations and/or genetic invasion may occur through a stepping‐stone mechanism (Hitt, Frissell, Muhlfeld, & Allendorf, 2003). This result highlights the need to potentially consider the influence of stocking at larger spatial scales that extend beyond the area of direct stocking effort.

Although we found strong evidence of minimal introgression, results from this study should be viewed circumspectly when assessing the potential risks of introgression from stocking. We did not evaluate possible declines in wild population fitness as a result of direct competition and/or failed mating attempts with stocked fish (Weber & Fausch, 2003; McGinnity et al., 2003). Likewise, we only evaluated neutral microsatellite markers and did not test for genetic introgression at loci linked to fitness (Harbicht, Alshamlih, et al., 2014; Harbicht, Wilson, et al., 2014) or at other areas of the genome where introgression may occur more readily (Ozerov et al., 2016). Evidence from other studies of stream salmonids suggests that rates of introgression can be high (Fleming & Petersson, 2001; Muhlfeld et al., 2009) and that it can lead to nonrandom changes to the genome that correlate to reductions in survival and fitness. As brook trout populations are often characterized by low levels of genetic diversity and small effective population sizes relative to other salmonids (Kelson et al., 2015; Ruzzante et al., 2016), they can be particularly susceptible to genetic invasion by captive populations as rates of introgression increases. Until a better understanding of the potential nongenetic effects of stocking are known and there is more investigation into the factors that influence rates of introgression, a cautionary approach to recreational stocking programs may still be warranted.

## DATA ARCHIVING STATEMENT

Data for this study are available from the Dryad Digital Repository: https://doi.org/10.5061/dryad.mb37t1q.
