# Supplementary material for: Limited hatchery introgression into wild brook trout (Salvelinus fontinalis) populations despite reoccurring stocking
Source: Evol Appl. 2018 Jun 14;11(9):1567–81. doi: 10.1111/eva.12646 (PMC6183464; doi:10.1111/eva.12646)
Supplement: Supplementary file 1 [file EVA-11-1567-s001.docx]

**Supporting Information**

**Methods**

We assessed the ability of our analytical methods to detect introgression in a simulated dataset with a known number of first-generation introgressed offspring. The majority of methods described below for centroid generation and individual assignment are analogous to those detailed in the main text, but are applied on two simulated populations.

The program EasyPop v 1.7 (Balloux, 2001) was used to create two populations, each consisting of 100 diploid individuals. Genotypic data consisted of 10 loci per individual, with 10 alleles per locus. For each population, there was a 1:1 sex ratio, migration and mutation parameters were set to 0, and recombination was allowed to occur. After 50 generations of random mating within each population, *F_ST_* between the two simulated population was 0.12, which is comparable to the observed average genetic distance between hatchery and wild populations in this study (average *F_ST_* = 0.13). These two simulated populations, each consisting of 100 individuals, are herein referred to as P1 and P2. We used HybridLab (Nielsen et al. 2006) to simulate random matings between P1 and P2 to create 10 introgressed individuals.

To create population centroids, we randomly selected a subset of 50 individuals each from P1 and P2, and used HybridLab to randomly mate those individuals 500 times. The 500 randomly mated individuals from P1 and 500 randomly mated individual from P2 constituted the two centroid populations. Individual assignment tests were performed in STRUCTURE v 2.3.4 (see main text for details), with a separate analysis for each individual from P1, P2, and the ten introgressed individual. Thus, each STRUCTURE analysis included the 500 individuals from the P1 centroid, the 500 individuals from the P2 centroid, and the genotype for one individual from either P1, P2, or one of the 10 introgressed individuals.

The STRUCTURE analysis produced each individual’s *P*(P1), the probability of assigning to the P1 centroid, which is analogous to *P*(wild) in the main text. To determine values of *P*(P1) commensurate with introgression, we developed a population of 500 introgressed individuals by using HybridLab to randomly mate individuals from the P1 and P2 50-indivdual subset used above for centroid generation. We completed independent STRUCTURE analyses on each of the 500 introgressed individuals, and the resulting 500 values of *P*(P1) constituted the expected distribution of *P*(P1) for introgressed individuals.

As in the main text, we used the 2.5 and 97.5 percentiles of the expected distribution of *P*(P1) as assignment thresholds (*i.e.,* values of *P*(P1) that fell between the 2.5 and 97.5 percentiles were used to classify an individual as introgressed). To determine the sensitivity of individual assignments to these thresholds, we compared results to individual assignments when using the less-conservative 5 and 95 percentiles of *P*(P1) as assignment thresholds.

**Results**

The expected distribution of *P*(P1) for an introgressed individual ranged from 0.31 to 0.69 using the 2.5 and 97.5 percentiles (Figure S1). This distribution changed to 0.35 to 0.65 when using the 5 and 95 percentiles (Figure S1). For the ten known introgressed individuals, values of *P*(P1) ranged from 0.33 to 0.67 (average *P*(P1) = 0.50).

All introgressed individuals were correctly assigned to introgressed origin when using the 2.5 and 97.5 percentiles as assignment thresholds. However, when using the 5 and 95 percentiles, one introgressed individual assigned to P2 and two introgressed individuals assigned to P2 (Figure S1). No ‘pure’ individuals were cross-assigned or to an introgressed origin in either scenario (*P*(P1) for P2= 0.93 to 1.00; *P*(P1) for P2 = 0.00 to 0.04).

**Literature Cited**

Balloux, F. (2001). EASYPOP (Version 1.7): A computer program for population genetics simulations. Journal of Heredity, 92(3), 301–302.

Nielsen, E. E., Bach, L. A., & Kotlicki, P. (2006). hybridlab (version 1.0): a program for generating simulated hybrids from population samples. Molecular Ecology Notes, 6(4), 971–973.

**Figure S1.** Histogram of *P*(P1) values for 500 introgressed individuals simulated by randomly mating individuals from P1 and P2. Blue lines correspond to the 2.5 and 97.5 percentiles and red lines to the 5 and 95 percentiles of the distribution. Values of *P*(P1) for the 10 known introgressed individuals are shown as points on x-axis.


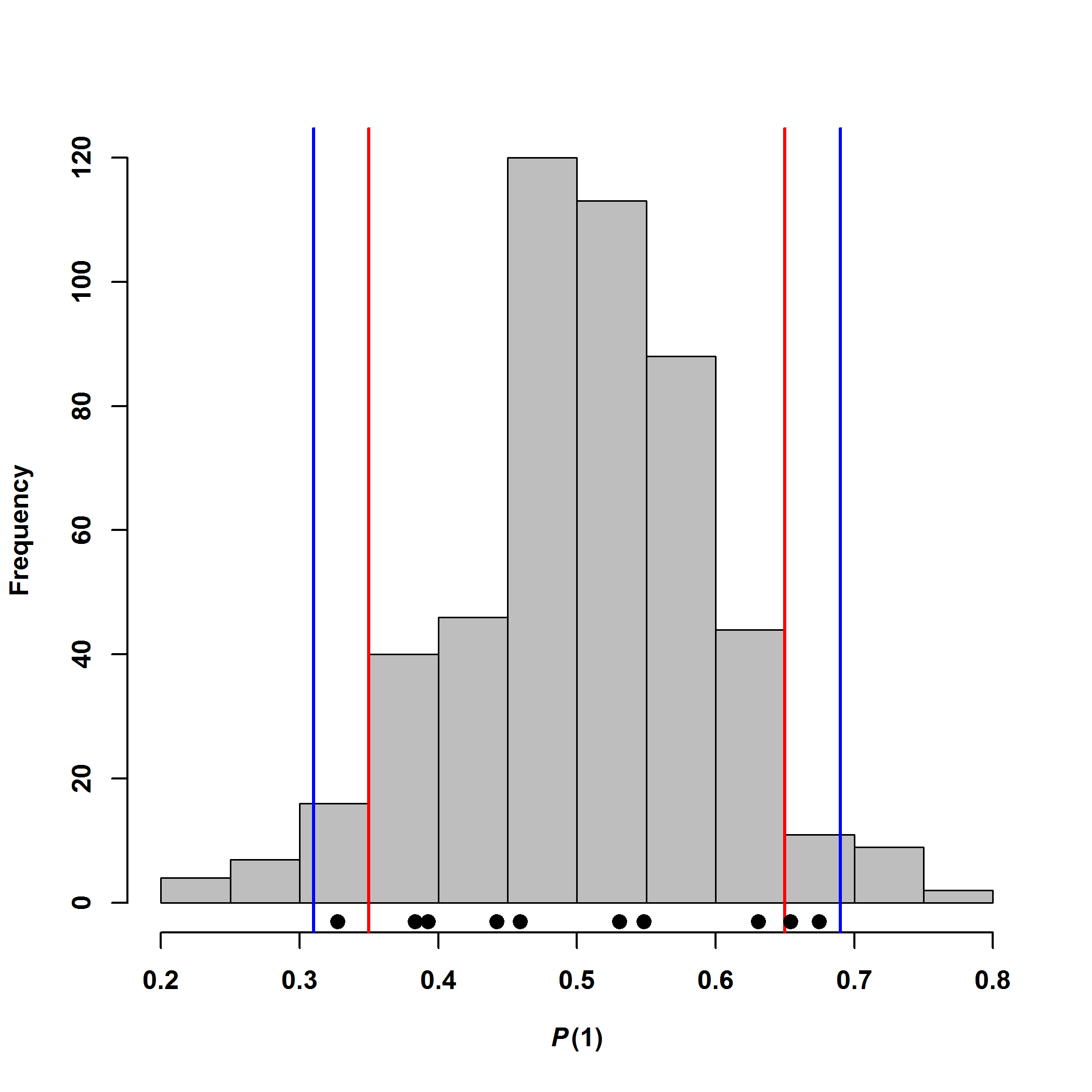


***P*(P1)**

Table S1: Sampling locations and stocking records for brook trout from 2006-2015 for 30 study sites in the Loyalsock Creek watershed. Numerical superscripts after site names indicate direct(1) or indirect(2) stocking, and lettered superscripts after site abbreviations group sites into sub-basins. Sites within the same sub-basin share the same indirect stocking source, and stocking densities among those sites are assumed identical. All fish stocked in the Loyalsock Creek watershed are from the Tylersville strain (TYL), a Benner Springs strain (BNSP) or from a PFBC Cooperative Fish Hatchery (PFBC-Coop).

| **Site Name** | **Abbr.** | **Lat** | **Lon** | **Stocking Period** | **Average Total Number of Fish Stocked Per Year (Range) Per Stream Section** | **Length of Stream Stocked (km)** | **Average Density (fish/km) of Fish Stocked Per Year** | | **Hatchery Source** | |  |
| --- | --- | --- | --- | --- | --- | --- | --- | --- | --- | --- | --- |
| Mill Run- Laporte | MILA | 41.47 | -76.48 | No record of stocking |  |  |  | |  | |  |
| Level Run | LEVL | 41.55 | -76.55 | No record of stocking |  |  |  | |  | |  |
| Mainstem Loyalsock Creek^1^ | MAIN16^a^ | 41.47 | -76.57 | 2009-2013 | 449.25  (282-750) | 11.30 | 39.76 | | TYL | |  |
| Upstream East Branch | EAST | 41.46 | -76.58 | No record of stocking |  |  |  | |  | |  |
| Unnamed Tributary to Elk Creek | UNT | 41.57 | -76.64 | No record of stocking |  |  |  | |  | |  |
| Bear Run^2^ | BEAR ^a^ | 41.47 | -76.50 | 2009-2013 | 449.25  (282-750) | 11.30 | 39.76 | | TYL | |  |
| Streby Run | STRB | 41.55 | -76.56 | No record of stocking |  |  |  | |  | |  |
| Huckle Run^2^ | HUCK^c^ | 41.42 | -76.71 | 2009, 2010, 2013, 2014 | 705.25  (376-1100) | 22.40 | 31.48 | | TYL | |  |
| Yellow Run^2^ | YELL^b^ | 41.51 | -76.5 | 2007,2009-2014 | 898.57  (470-1405) | 7.10 | 126.56 | | TYL, BNSP | |  |
| Sand Spring Run^2^ | SSR^i^ | 41.34 | -76.8 | 2006-2015 | 3491.22  (2435-4100) | 6.60 | 528.97 | | TYL, PFBC-Coop | |  |
| Grandad Run | GRAN | 41.36 | -76.75 | No record of stocking |  |  |  | |  | |  |
| Red Run^2^ | RED^i^ | 41.35 | -76.81 | 2006-2015 | 3491.22  (2435-4100) | 6.60 | 528.97 | | TYL, PFBC-Coop | |  |
| Pole Bridge Run^1,2^ | POLE ^a^ | 41.45 | -76.53 | ^1^2006-2011 | 365.83  (295-700) | 4.00 | 91.46 | | TYL | |  |
|  |  |  |  | ^2^2009-2013 | 449.25  (282-750) | 11.30 | 39.76 | | TYL | |  |
| Scar Run^2^ | SCAR^c^ | 41.47 | -76.64 | 2009, 2010, 2013, 2014 | 705.25  (376-1100) | 22.40 | 31.48 | | TYL | |  |
| Downstream East Branch^2^ | DSEA^j^ | 41.46 | -76.58 | 2006-2009 | 323.75  (295-400) | 3.01 | 107.56 | | TYL | |  |
| Shanerburg Run^1,2^ | SHAN ^a^ | 41.45 | -76.53 | ^1^2006-2011 | 265.83 (95-300) | 2.40 | 110.76 | | TYL | |  |
|  |  |  |  | ^2^2009-2013 | 449.25  (282-750) | 11.30 | 39.76 | | TYL | |  |
| Coal Run^2^ | COAL^a^ | 41.46 | -76.52 | 2009-2013 | 449.25  (282-750) | 11.30 | 39.76 | | TYL | |  |
| Brunnerdale Run | BRUN | 41.39 | -76.67 | No record of stocking |  |  |  | |  | |  |
| Rock Run^2^ | ROCK^b^ | 41.5 | -76.52 | 2007,2009-2014 | 898.57  (470-1405) | 7.10 | 126.56 | | TYL, BNSP | |  |
| Snake Run^2^ | SNAK^g^ | 41.38 | -76.78 | 2009-2014 | 1473.33  (1045-2130) | 19.30 | 76.34 | | TYL | |  |
| Weed Run^2^ | WEED^e^ | 41.53 | -76.74 | 2006-2015 | 1636.00  (188-3460) | 6.50 | 251.69 | | TYL | |  |
| Dry Run- Hoagland Branch^2^ | DRHO^e^ | 41.51 | -76.71 | 2006-2015 | 1636.00  (188-3460) | 6.50 | 251.69 | | TYL | |  |
| Jacoby Hallow^2^ | JACO^h^ | 41.37 | -76.92 | 2006-2008,2010,2011,2014 | 466.67  (250-650) | 12.81 | 36.42 | | PFBC-Coop | |  |
| Lick Run^2^ | LICK^d^ | 41.51 | -76.64 | 2006,2007,2009-2013,2015 | 151.50  (105-300) | 4.73 | 32.03 | | TYL, BNSP | |  |
| Double Run^1,2^ | DOUB^a^ | 41.47 | -76.58 | ^1^2006-2009 | 323.75  (295-400) | 3.01 | 107.56 | | TYL | |  |
|  |  |  |  | ^2^2009-2013 | 449.25  (282-750) | 11.30 | 39.76 | | TYL | |  |
| Flag Marsh Run | FLAG | 41.48 | -76.31 | Not directly stocked and records for downstream confluence are unavailable | | | | | | |  |
| Mill Creek- Hillsgrove^1^ | MIHI^f^ | 41.48 | -76.75 | 2006-2015 | 301.70  (235-400) | 4.60 | 65.59 | | TYL | |  |
| Conklin Run | CONK | 41.42 | -76.48 | No record of stocking |  |  |  | |  | |  |
| Swamp Run^2^ | SWAM^e^ | 41.49 | -76.72 | 2006-2015 | 1636.00  (188-3460) | 6.50 | 251.69 | | TYL | |  |
| Little Bear Creek^1,2^ | DSLB^g^ | 41.36 | -76.84 | ^1^2006-2015 | 3491.22  (2435-4100) | 6.60 | 528.97 | | TYL, PFBC-Coop | |  |
|  |  |  |  | ^2^2009-2014 | 1473.33  (1045-2130) | 19.30 | 76.34 | | TYL | |  |
| ^1^ Stocking occurs at the sample location | | | | |  |  |  | |  | |  |
| ^2^ Stocking within 2km of the sample location | | | | | |  | |  | |  | |

| **Scale of the Predictor** | **Environmental Variable** | **Abbreviation** | **Maximum** | **Minimum** | **Average** |
| --- | --- | --- | --- | --- | --- |
| Watershed-level Covariates | Percent watershed forested | Tree | 1.00 | 0.45 | 0.90 |
|  | Watershed Area (km^2^) | Area | 19.66 | 1.86 | 6.47 |
|  | Shortest Distance to a Stocked Reach (km) | ShortStock | 10.10 | 0.00 | 2.13 |
| Site-level Covariates | Temperature (°C) | Temp | 14.87 | 10.90 | 13.32 |
|  | pH | pH | 7.36 | 4.66 | 6.61 |
|  | Conductivity ( mho/cm ) | Cond | 156.30 | 12.97 | 50.10 |
|  | Total Alkalinity (mg/L) | TotAlk | 42.10 | 0.57 | 13.58 |
|  | Dissolved Oxygen | DO | 10.39 | 6.45 | 9.47 |
|  | Hardness | Hard | 48.00 | 1.00 | 15.20 |
|  | Average Stream Width | Width | 4.05 | 1.50 | 2.65 |
|  | Density of Adult Brook Trout (per 100 m^2^) | Den | 41.61 | 6.03 | 16.44 |

Table S2. Environmental covariates (including the maximum, minimum, and average across the 30 sample sites), used to model introgression in response to watershed-level and site-level predictors. Site-level covariates represent average values from 2013 to 2015. Covariate abbreviations are used in Table S3 when describing model composition and performance. Percent of the watershed that is forested was removed from consideration in all models because all but one site was >90% forested. Additionally, total alkalinity and hardness were removed because of significant correlations with conductivity, and a correlation between watershed area and stream width precluded use of both terms in a single model.

Table S3. Descriptions of the four hierarchical logistic regression models used to determine the effects of watershed-level and site-level predictors on introgression. All models include site as a random effect to account for multiple individuals collected from each sample location. Models covariates included shortest distance to a stocking location (ShortStock), watershed area (Area), stream temperature (Temp), conductivity (Cond), pH, dissolved oxygen (DO), adult brook trout density (Den), and average stream width (Width). Models were assessed using Akaike’s information criterion for small sample sizes (AIC_C_), including ΔAIC_C_ (the differences between the value for the i^th^ model and the minimum AIC_C_ value for all models) and *w_i_*, the weight for the i^th^ model.

| Model Name | Model Covariates | AIC_C_ | ΔAIC_C_ | *w_i_* | R^2^ |
| --- | --- | --- | --- | --- | --- |
| Watershed-level Model | ShortStock, Area | 409.35 | 0.00 | 0.48 | 0.001 |
| Site-level Model | Temp, Cond, pH, DO, Den, Width | 409.70 | 0.35 | 0.40 | 0.028 |
| Multi-scale Model | ShortStock, Temp, Cond, pH, Area, DO, Den | 413.19 | 3.84 | 0.07 | 0.019 |
| Literature-supported Model | ShortStock, Area, pH, DO, Temp, Den | 413.99 | 4.64 | 0.05 | 0.010 |

| Parameter | Estimate ± SE | Lower 95% CI | Upper 95% CI |
| --- | --- | --- | --- |
| ShortStock | -0.02 ± 0.25 | -0.50 | 0.47 |
| Area | -0.10 ± 0.26 | -0.60 | 0.40 |
| Cond | -0.34 ± 0.47 | -1.27 | 0.58 |
| pH | -0.16 ± 0.27 | -0.68 | 0.36 |
| Temp | 0.38 ± 0.49 | -0.58 | 1.34 |
| DO | 0.03 ± 0.20 | -0.36 | 0.41 |
| Den | -0.11 ± 0.24 | -0.58 | 0.35 |
| Width | -0.33 ± 0.43 | -1.17 | 0.51 |

Table S4. Parameter estimates (± 1 standard error) with upper and lower 95% confidence intervals from the consensus model representing the average of the site-level and watershed-level models. The multi-scale and literature-supported models were not included in the average model as they had AIC_C_ > 2.
